# LsARF3 mediates thermally induced bolting through promoting the expression of *LsCO* in lettuce (*Lactuca sativa* L.)

**DOI:** 10.3389/fpls.2022.958833

**Published:** 2022-09-08

**Authors:** Yunfeng Li, Jiaqi Zhu, Yixuan Feng, Zhenfeng Li, Zheng Ren, Ning Liu, Chaojie Liu, Jinghong Hao, Yingyan Han

**Affiliations:** ^1^Beijing Key Laboratory of New Technology in Agricultural Application, National Demonstration Center for Experimental Plant Production Education, Plant Science and Technology College, Beijing University of Agriculture, Beijing, China; ^2^National Engineering Research Center for Vegetables, Institute of Vegetable Science, Beijing Academy of Agriculture and Forestry Sciences, Beijing, China

**Keywords:** lettuce, bolting, Auxin response factor, LsARF3, *LsCO*, overexpression, knockout

## Abstract

Lettuce (*Lactuca sativa* L.) is a leafy vegetable whose edible organs usually are leaf or stems, and thus high-temperature induced bolting followed by flower initiation is an undesirable trait in lettuce production. However, the molecular mechanism that controls lettuce bolting and flowering upon thermal treatments is largely unknown. Here, we identified a Lettuce *auxin response factor 3* (*LsARF3*), the expression of which was enhanced by heat and auxin treatments. Interestingly, *LsARF3* is preferentially expressed in stem apex, suggesting it might be associated with lettuce bolting. Transgenic lettuce overexpressing *LsARF3* displayed early bolting and flowering, whereas knockout of *LsARF3* dramatically delayed bolting and flowering in lettuce under normal or high temperature conditions. Furthermore, Exogenous application of IAA failed to rescue the late-bolting and -flowering phenotype of *lsarf3* mutants. Several floral integrator genes including *LsCO*, *LsFT*, and *LsLFY* were co-expressed with *LsARF3* in the overexpression and knockout lettuce plants. Yeast one-hybrid (Y1H) experiments suggested that LsARF3 could physically interact with the *LsCO* promoter, which was further confirmed by a dual luciferase assay in tobacco leaves. The results indicated that LsARF3 might directly modulate the expression of *LsCO* in lettuce. Therefore, these results demonstrate that *LsARF3* could promote lettuce bolting in response to the high temperature by directly or indirectly activating the expression of floral genes such as *LsCO,* which provides new insights into lettuce bolting in the context of ARFs signaling and heat response.

## Introduction

Lettuce (*Lactuca sativa* L.) is a popular leafy vegetable which is harvested and consumed during its vegetative growth. Thus, early bolting can result in a significant reduction in crop yield and culinary quality, and consequently is an undesirable trait in lettuce cultivation and breeding (Simonne et al., 2002; Han et al., 2021). Bolting is characterized as the elongation of the floral stem, accompanied with the initiation of the flowering (Pouteau and Albertini, 2009). It has been proposed that bolting and flowering are regulated by an elaborate network of genetic pathways that incorporate endogenous and environmental cues, such as vernalization, photoperiod, circadian clock, gibberellin, nutrient supply, and ambient temperature (Andrés and Coupland, 2012; Kinoshita and Richter, 2020; Yan et al., 2021). The molecular basis of photoperiodic induction of floral transition has been studied intensively in the model plant *Arabidopsis* (Blümel et al., 2015). It has been demonstrated that the bolting and flowering signals from internal or external factors usually converge on a few integrator genes which serve as flowering inducers or repressors during floral transition. Floral inducers including FLOWERING LOCUS T (FT), FD, SUPPRESSOR OF CONSTANS 1 (SOC1), and LFEAY (LFY) activate the transcription of the majority of genes floral genes, promoting *Arabidopsis* bolting and flowering (Johansson and Staiger, 2015). Besides, nuclear proteins CONSTANS (CO) and GIGANTEA (GI) also promote flowering through photoperiod and circadian pathways, making them master regulators affecting floral transition (Valverde, 2011; Brandoli et al., 2020). On the contrary, transcription factor FLOWERING LOCUS C (FLC) is the major flowering repressor, which antagonizes gibberellin and photoperiod pathways and delays bolting and flowering (Sheldon et al., 2000). *Arabidopsis flc* mutant exhibits accelerated flowering independent of vernalization, whereas some accessions with functional *FLC* genes do need vernalization for summer flowering (Shen et al., 2022). Although progresses have been achieved in the photoperiod and vernalization, our knowledge of the effects of ambient temperature on flowering and bolting remains intangible.

It is well-known that ambient temperature has pronounced, albeit complex, effects on bolting and flowering time (Mcclung et al., 2016). In *Arabidopsis*, bolting and flowering time are strongly influenced by high temperatures (Andrés and Coupland, 2012; Takeno, 2016). Previous studies suggested that, when exposed to high growth temperature, *Arabidopsis* can accelerate its floral transition and seed production to survive the adverse environment. Similarly, most lettuce cultivars prefer to grow in cool conditions (16°C–18°C), while heat temperature (>24°C, may be variable depending on the genetic background) promotes lettuce bolting and flowering (Katz and Weaver, 2003; Yamori et al., 2022). Some lettuce cultivars such as GB-30 are also extremely sensitive to the thermal treatments and can initiate their floral stem elongation, as well as flowering, within one-week growth at a greenhouse with the temperature above 33°C (Hao et al., 2021), thus leading to severe economic loss to lettuce growers, especially under frequently occurred heat waves in summers. Thus, investigations on heat-induced flowering are of great interest to biologists as well as breeders.

In *Arabidopsis*, at least two bHLH transcription factors PHYTROCHROME INTERACTING FACTOR4 (PIF4) and PIF5 promote flowering through the induction of FT and its paralog TWIN SISTER OF FT (TSF) during thermally induce flowering (Kunihiro et al., 2011; Kumar et al., 2012; Mcclung et al., 2016). Furthermore, PIF4 can physically interact with CO and other components to form a complex, thereby contributing to the upregulation of FT and TSF (Kumar et al., 2012). These results indicate that “heat signals” are relayed to FT and/or its homologs, upregulation of which contributes to the acceleration of bolting and flowering in response to elevated temperature. Studies in lettuce suggested that heat shock transcription factors (HSFs) might be involved in the floral induction pathway, and its binding motifs were identified in the promoter regions of LsSOC1 and LsMADS55, putative lettuce orthologues to *Arabidopsis* SOC1 and APETALA1(Chen et al., 2018; Ning et al., 2019). Knockdown or RNA inference lines of LsSOC1 are insensitive to high temperature, and overexpression of LsMADS55 resulted in early flowering in transgenic *Arabidopsis* lines, suggesting that their important roles in heat-promoted bolting and flowering (Chen et al., 2018; Ning et al., 2019). Thus, it has been postulated that heat-accelerated floral transition in lettuce might be regulated by a number of HSF transcription factors (Chen et al., 2018; Ning et al., 2019). Proteomic analysis revealed that HEAT SHOCK PROTEIN 70 (HSP70) is differentially expressed between heat-sensitive and heat-tolerate lettuce cultivars, further supporting the possibility of the HSP-modulated flowering pathway in lettuce (Li et al., 2017). Another study compared the transcriptome of heat-treated (bolted) and control (non-bolted) lettuce and found that C2H2 zinc finger, AP2/EREBP, and WRKY transcription factors might be associated with heat-accelerated bolting (Liu et al., 2018). Although there is no experimental evidence that PIFs were involved in thermally induced floral transition in lettuce, current findings suggested that the floral transition in lettuce might be modulated under different regulatory pathways from that of *Arabidopsis*.

Auxin is a key phytohormone that regulates many physiological and developmental processes in plants, such as embryogenesis, organogenesis, directional growth, and organ structure (Enders and Strader, 2015). Auxin response factors (ARFs) represent the core component of the auxin signal cascade, as they are responsible for the transcriptional regulation of a defined set of genes in the auxin pathway (Roosjen et al., 2018). Most ARF protein contains a conserved amino-terminal DNA-binding domain (DBD) responsible for the binding to TGTCTC motif, a variable middle region that confers activator or repressor activity, and a C-terminal PB1 (formally called IAA/Aux domain) involved in homo- and hetero-dimerization (Liu et al., 2014a; Li et al., 2016; Roosjen et al., 2018). However, *Arabidopsis* ARF3/ETTIN (ETT) encodes a truncated ARF proteins which lack PB1 domains (Nemhauser et al., 2000; Guilfoyle and Hagen, 2007), and thus ARF3 could be insensitive to auxin. Nevertheless, ARF3 functions in some auxin-involved pathways, including polarity specification, gynoecium patterning, floral meristem maintenance and termination, and early flower development, in *Arabidopsis* (Nemhauser et al., 2000; Liu et al., 2014b; Simonini et al., 2017; Kuhn et al., 2020). For example, ARF3 controls floral meristem determinacy by directly repressing the expression of *ISOPENTENYLTRANSFERAS*E (*IPT*) family genes (Cheng et al., 2013). ARF3 is a direct target of APETALA2 (AP2), which is associated with its role in floral meristem determinacy (Liu et al., 2014b). Transcription of *ARF3* itself is repressed by TAS3-derived trans-activating small interfering RNAs (tasiRNAs), which ensures leaf polarity in *Arabidopsis* (Hunter et al., 2006). Noteworthy, a recent advance showed that a number of drought stress-related genes were upregulated in *ARF3* overexpressing *Arabidopsis*, indicating that *ARF3* is important for those genes’ expression under dehydration stress (Zheng et al., 2018). Therefore, it can be concluded that ARF3 is emerging as a key regulator of abiotic stress and development in *Arabidopsis*. However, how ARF3 participates in the accelerated floral transition under thermal treatments remains to be elucidated.

Our previous study showed that the majority of LsARFs were positively respond to heat stress and lettuce bolting (Qi, 2019; Wang et al., 2022b). Of these heat-inducible *LsARF* genes, *LsARF3* expression was stimulated by elevated temperatures and maintained higher expression level with the progression of heat stress, indicating LsARFs might be implicated in the heat-accelerated floral transition (Qi, 2019). However, the molecular mechanism by which ARF3 regulates thermally induced bolting and flowering is still unclear. In this study, we demonstrate that the expression level of *LsARF3* was increased upon the floral transition and thermal treatments. Transgenic lettuce overexpressing *LsARF3* exhibited early bolting and flowering phenotypes. By contrast, the floral transition of lettuce *lsarf3* mutants was delayed under thermal treatment, and exogenous application of auxin failed to rescue the late bolting of *lsarf3* mutants. We also showed that several floral genes such as *LsCO*, *LsFT*, and *LsLFY*, might be directly regulated by *LsARF3* through co-expression and Yeast one-hybridization analysis. Those findings not only identify a new player in thermally accelerated bolting but also contribute to a better understanding of ARFs signaling and heat response in lettuce.

## Materials and methods

### Plant materials and treatment

Lettuce (*Lactuca sativa* L.) of GB-30, a variety, which bolts easily and comes from seeding plant protection Co., LTD in Inner Mongolia Bameng Fidelity, were conserved in our laboratory. Seeds were sown in a mixture of sand, soil, and peat (volume ratio of 1:1:1), and grown in the growth chamber under normal conditions (20°C/13°C (day/night), 14 light and 10 h darkness, and 60% relative humidity). Standard pest control and water management practices were used. The seedlings were transplanted into 6 cm × 8 cm pots at the trefoil stage. When the plants developed the sixth true leaf, they were divided into two groups. The control group continued growing under the normal growth conditions as described above. The other group was moved to another growth chamber and treated with high temperatures of 33°C and 25°C during the day and night, respectively. The other environmental factors were unchanged. After high-temperature treatment for 0, 2, 4, 6, 8, 10, 12, 24 h and 0, 8, 16, 24 days, shoot apex of lettuce were harvested for RNA extractions. Root, stem (stalk), stem apex, leaf, flower, fruit, and seed tissue samples from lettuce grown under the normal temperature conditions were also collected. All samples were immediately frozen in liquid nitrogen and stored at −80 ^°^ C for further measurements on gene expression analysis. Every five plants were used as one replicate for three replicates.

### RNA extraction, synthesis of cDNA, *LsARF3* cloning, and bioinformatics analysis

Total RNA was extracted from specific tissues using the Quick RNA isolation Kit (Huayueyang Biotech, Beijing, China), following the user manual from the manufacturer. NanoDrop 2000 Spectrophotometer (Thermo Fisher Scientific, Wilmington, DE, United States) and 1% agarose gel electrophoresis were used to determine RNA quality and concentration. The extracted RNA was digested with EasyScript® One-Step gDNA Removal and cDNA Synthesis SuperMix (Transgen, Beijing, China) was used to synthesize cDNA.

The open reading frame of the cDNA was amplified with gene-specific primers of *LsARF3-*F/R (Supplementary Table1) based on *LsARF3* (AccessionXM_023880987.2) available at NCBI. The whole cDNA of *LsARF3* was then amplified with mixed super fidelity polymerase (Vazyme Biotech Co., Ltd.), under thermal cycling conditions of 95°C denaturation for 3 min, followed by 35 cycles of 95°C for 15 s, 60°C for 15 s, 72°C for 2 min, and a final extension of 72°C for 5 min. A single band of expected size 1794 bp was purified from gel and sub-cloned into pEASY vector (TransGen Biotech, Beijing, China).

A homology search of sequences was carried out through BLAST in NCBI, and multiple alignments were analyzed using DNAMAN 9.0. A phylogenetic tree was constructed using the neighbor-joining method (NJ) with MEGA 7.0 software.

### Quantitative real-time PCR

The total RNA of fresh lettuce samples was extracted by quick RNA Isolation Kit (Huayueyang, China), and the cDNA was obtained by transcript one step gDNA removal and cDNA synthesis Supermix reagent (Transgen, China). Then RR430b TB green fast qPCR mix (Takara, Japan) preparation system, and the fluorescence data were obtained by Bole bio rad CFX96 real-time fluorescence quantitative PCR instrument. 18 s rRNA (HM047292.1) was selected as the internal reference gene, and the internal reference gene and samples were biologically repeated three times and technically repeated three times (Supplementary Table1). The relative gene expression is based on the 2^−Δ ΔCt^ method.

### Subcellular localization

*pRI101* Carrying GFP label after transformation was selected, and *pRI101*-*LsARF3* vector was constructed through homologous recombination. The 35S promoter was used for directing the expression of the fusion gene. Subcellular localization of *LsARF3* by fusion with a green fluorescent protein (GFP) in the C-terminal region. It was injected into tobacco with five leaves by Agrobacterium infection. After dark culture for 2 days, it was observed and photographed under a laser confocal microscope.

### Overexpression and CRISPR/Cas9 plasmid construction

Using *LsARF3* clone plasmid as a template, primer *LsARF3*-OE-F/R (Supplementary Table1), amplify the target band and add a homologous arm. NdeI restriction endonuclease single enzyme digested the expression vector *pRI101-GFP* to linearize the plasmid. Clonexpress II one-step cloning kit homologous recombinant PCR product and linearized plasmid were transferred to DH5α competent cell. After colony PCR, the positive clones were sent to sequencing, and the correct sequenced plasmid was transferred into Agrobacterium GV3101. The sequencing primer was pRI101-F/R (Supplementary Table1).

According to the principle of adjacent motif recognition (PAM) of the original spacer sequence of the CRISPR/Cas9 system and the design principle of guide RNAs (gRNAs) of gene knockout target site, CRISPR gRNA is used to design the website^1^ and NCBI-Blast potential knockout site detection, and select the two most suitable gRNAs. Four primers were designed according to two gRNA sequences. CRISPR/Cas9 plasmid *pKSE401-DT1T2* carries two tandem repeat gRNA expression boxes and Cas9 enzyme. Using *pCBC-DT1T2* plasmid as a template, four primers DT1-BsF + DT1-F0/DT1-BsR + DT1-R0 (Supplementary Table1) were amplified to obtain a tandem repeat expression box fragment carrying gRNA1 and gRNA2 with homologous arm. *Bsai-HFv2* restriction enzyme linearized *pKSE401* vector, ClonExpress II One Step Cloning Kit homologous recombinant PCR product and linearized plasmid, and transferred to DH5α competent cell. After colony PCR, the positive clones were sent for sequencing. The plasmids in which both two gRNAs were successfully constructed were transferred into Agrobacterium GV3101. The colony PCR and sequencing primers were U626-IDF/U629-IDR (Supplementary Table1).

### Genetic transformation of lettuce

To generate the *LsARF3* transgenic lettuce, Agrobacterium-mediated leaf disk transformation of lettuce. Cotyledons of 4-day-old seedlings were pre-cultured in an MS medium supplemented with 0.5 mg/l 6-BA, and 0.05 mg/l NAA for 1 day. The cotyledons were then immersed for 5 min in an Agrobacterium culture (OD600 = 0.1–0.3) with the liquid MS medium. The cotyledons were dried and then co-cultured for 30 h in an MS medium containing 0.5 mg/l Kinetin, and 0.05 mg/l NAA under dark conditions. After co-cultivation, the cotyledons were transformed into a regeneration and selection MS medium (0.5 mg/l 6-BA, 0.05 mg/l NAA, 300 mg/l Timentin, 100 mg/l Kanamycin). After every week, the medium was refreshed until the shooting. Two-to-three-centimeter newly developed shoots were then transformed into a rooting MS medium (0.05 mg/l NAA and 300 mg/l Timentin) until true roots developed. Once the regenerated seedlings had enough and strong roots, they were transplanted to nutrient soil and continued to grow in the greenhouse.

The identification method of overexpression positive seedlings is as follows: T_0_ takes the leaves of transgenic seedlings to extract DNA, takes DNA as template, pRI101-F/R as identification primer, amplifies bands, and those with clear bands are positive seedlings. After the seeds of T_0_ generation positive seedlings were harvested and sown, the leaves of T_1_ generation transgenic seedlings were taken to extract RNA and reverse transcribed to synthesize cDNA. The cDNA was used as the template and GFP-F/R (Supplementary Table1) was used as the identification primer to amplify the bands. If the expression increased significantly, it was a positive seedling. Through the above methods, we have obtained three T_1_ lines of overexpressed *LsARF3*. The identification method of gene editing positive seedlings is as follows: take the leaf DNA of transgenic seedlings as the template, U626-IDF/U629-IDR and Cas9-F/R as the primers, and those with two bright bands in amplification are transgenic positive seedlings. On the genomic DNA sequence of leaf lettuce, the primers CRISPR-DNA-F/R (Supplementary Table1) were designed at about 200 bp before and after the two gRNAs to amplify the target sequence of positive seedlings, and the PCR products were sent to sequencing to identify the editing type. Unedited and homozygous editing types can be directly determined by the sequencing results of PCR products. If the sequencing results of PCR products are bimodal, it is necessary to connect the PCR products to the Blunt-Zero cloning vector, and send at least 10 monoclonal antibodies to each sample for sequencing. The results were consistent and could be regarded as positive seedlings. Through the above methods, three homozygous T_1_ lines of *LsARF3* gene editing were obtained.

The phenotypic changes of *LsARF3*-OE and WT plants were observed and recorded at normal temperature (NT), the stem length changes were measured every day. The *lsarf3* mutants and WT plants were treated with high temperature (HT) when they had six leaves. After treatment, the changes in phenotype and stem length were observed and recorded every day. In both *LsARF3*-OE and *lsarf3* mutants, when the stem length was significantly different compared to WT plants, flower bud differentiation of stem apexs were observed regularly by paraffin section method (Zhang et al., 2016) to determine bolting time.

### Exogenous IAA application

When the seedlings of *lsarf3* mutants and WT plants grow to six leaves, they are sprayed every other week at a concentration of 40 mg/Kg for four consecutive weeks. The plant phenotype was observed and photographed every day, the stem length was measured every 8 days, the progress of flower bud differentiation of stem apex was determined by the paraffin section method (Zhang et al., 2016), and the transcript level of *LsARF3* was detected by qRT-PCR at 24 days after exogenous IAA application.

### Yeast one-hybrid assay

For the Yeast one-hybrid (Y1H) screening assay, the promoter of *LsCO* was amplified with specific primers (Supplementary Table 1). *LsCO* promoter was cloned into the *pHIS2* vector, and the full-length coding region of *LsARF3* was ligated into the *pGADT7* vector, respectively. The combined pHIS2 vector was linearized and transformed to Y187 yeast strain together with an empty AD vector or *pGADT7-LsARF3*. The transformed yeast cells were screened on SD/−Leu-Trp-media and SD/−Leu-Trp-His + 60 mM 3AT-medium.

### Dual-luciferase assay

Dual-luciferase assay was performed according to the previous study (Ma et al., 2019). The *LsCO* promoter (2,000 bp) was cloned into the pGreenII 0800-LUC vector at the PstI and *BamHI* restriction enzyme sites. The *LsARF3* coding sequence was cloned into the pGreenII 62-SK vector. For the co-expression analysis, leaves from 5- to 6-week-old tobacco plants were transformed with the recombinant plasmids; the no-effector construct was used as a negative control. The Dual-Luciferase® Reporter Assay System (Promega, Bei-jing, China) was used to analyze firefly luciferase (LUC) and *Renilla* luciferase (REN) activities. The LUC:REN ratio was used to determine activation or repression.

### Statistical analysis

Three independent biological replicates sampled from different plants were used for each determination. Data were presented as means ± standard errors (SEs) of three replicates. Statistical analysis of the bioassays were performed with SPSS statistical software (version 19.0, SPSS Inc., Chicago, IL, United States). Student’s *t*-test was used to test for significance at levels of *p* < 0.05 (∗) or *p* < 0.01 (∗∗).

## Results

### Identification and expression analysis of *LsARF3* gene in lettuce

**Figure 1:**
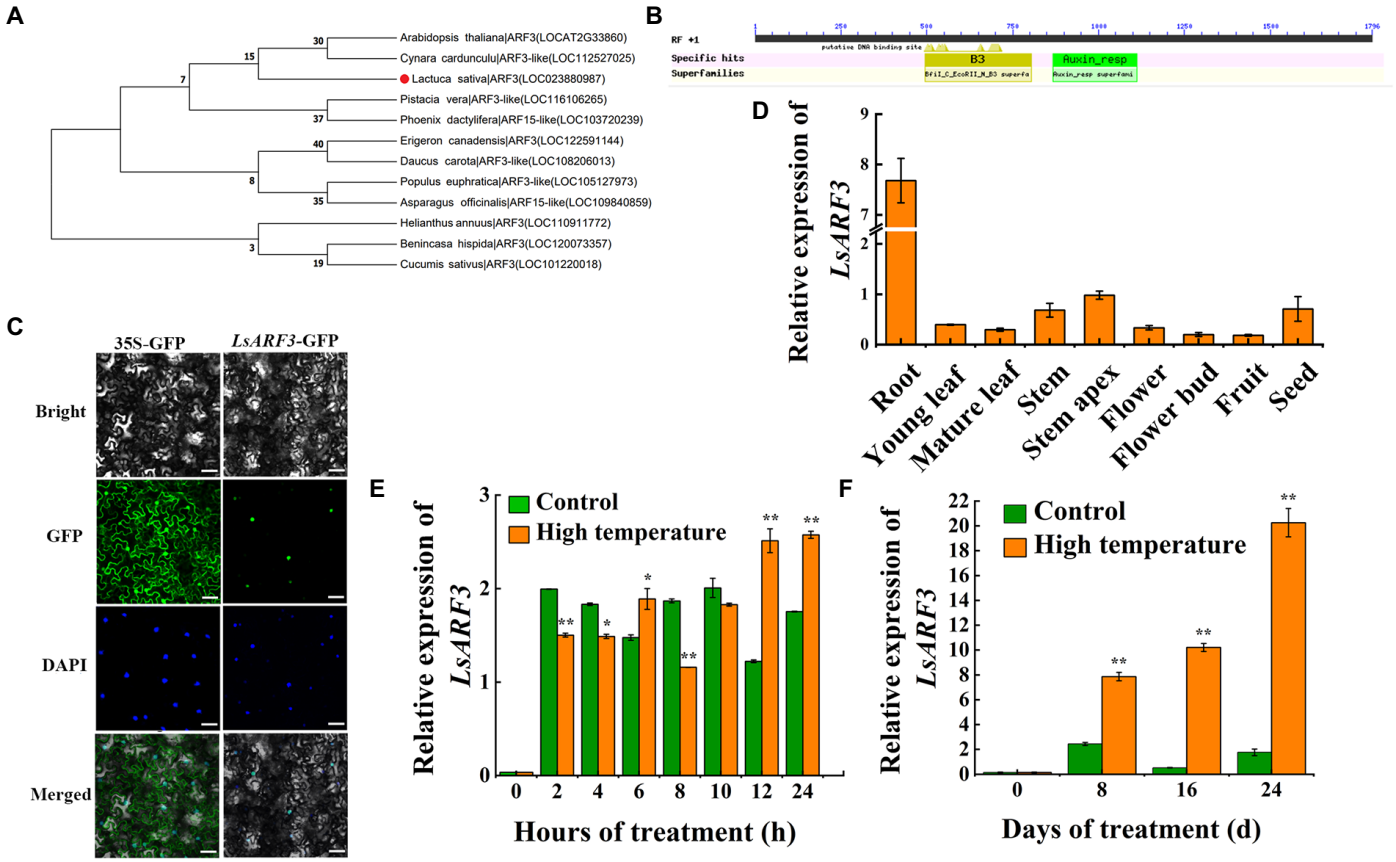
Identification and expression analysis of *LsARF3* in lettuce. **(A)** Phylogenetic tree of LsARF3 protein. **(B)** Analysis of conserved domains of LsARF3 protein in lettuce. **(C)** Subcellular localization of LsARF3 fusion protein in lettuce cells. Plasmid with green fluorescent protein (GFP) alone served as the control. Bar = 50 μm. **(D)** Expression level of *LsARF3* in different organs of lettuce. Gene encoding 18 s rRNA was selected as the internal control. **(E)** Expression level of *LsARF3* in stem apex within 24 h of high temperature stress. **(F)** Expression level of *LsARF3* in stem apex within 24 days of high temperature stress. Significant differences were tested by Student’s *t*-test (^*^indicates *p* < 0.05 and ^**^indicates *p* < 0.01).

To explore the roles of the ARF3 transcription factor on lettuce bolting, LsARF3 was identified through a BLAST search against lettuce genome using Arabidopsps ARF3 (AtARF3) as query sequence. Based on phylogenetic relationships, we found that Lsat_1_v5_gn_4_51680 shared high sequence homology with AtARF3 proteins as well as other ARF3 reported in 11 plant species such as artichoke, populus, asparagus, and etc. Phylogenetic analysis of all these ARF3 proteins showed that they could be divided into two groups (Figure 1A). Among these ARF3s, members from lettuce, *Arabidopsis*, and artichoke were grouped into the first subclade, suggesting their close relationship; ARFs from poplars, cucumbers, and other plant species consist the second group. Analysis of the conserved domains of LsARF3 protein suggested that the LsARF3 had two conserved domains: B3, and Auxin_resp (Figure 1B). The B3 domain is the motif the is responsible for the binding to target DNA regions, while Auxin_resp motif is the conserved domain specific to the ARFs. Combined with the sequence alignments and phylogenetic analysis, it can be inferred that LsARF3/Lsat_1_v5_gn_4_51680 is the lettuce orthologs of the AtARF3, which might encode the ARF3 transcription factors.

The subcellular localization results suggest that LsARF3 is a nuclear protein (Figure 1C), which is consistent with its roles as transcription factor. In the analysis of tissue/organ-specific expression, *LsARF3* had a relatively higher level of expression at lettuce root, stem apex, and seed (Figure 1D), indicating that its functions might be associated with the meristem of the stem apex and root apex. After high-temperature treatment, the expression level of *LsARF3* gene decreased in the early stage and increased in late stage with significant difference within 24 h (Figure 1E). Furthermore, within 24 days of high-temperature treatment, the *LsARF3* gene in the stem apex was significantly higher, compared to the control group, and the increasing trend was more significant with the extended treatment time (Figure 1F), further suggesting that *LsARF3* expression was induced by high temperature. Since our previous study showed that high temperature promotes the bolting of lettuce (Hao et al., 2018), It is reasonable that LsARF3 might be involved in the regulation of accelerated bolting under high temperature.

### CRISPR/Cas9-mediated knockout of *LsARF3* delays bolting of lettuce

**Figure 2:**
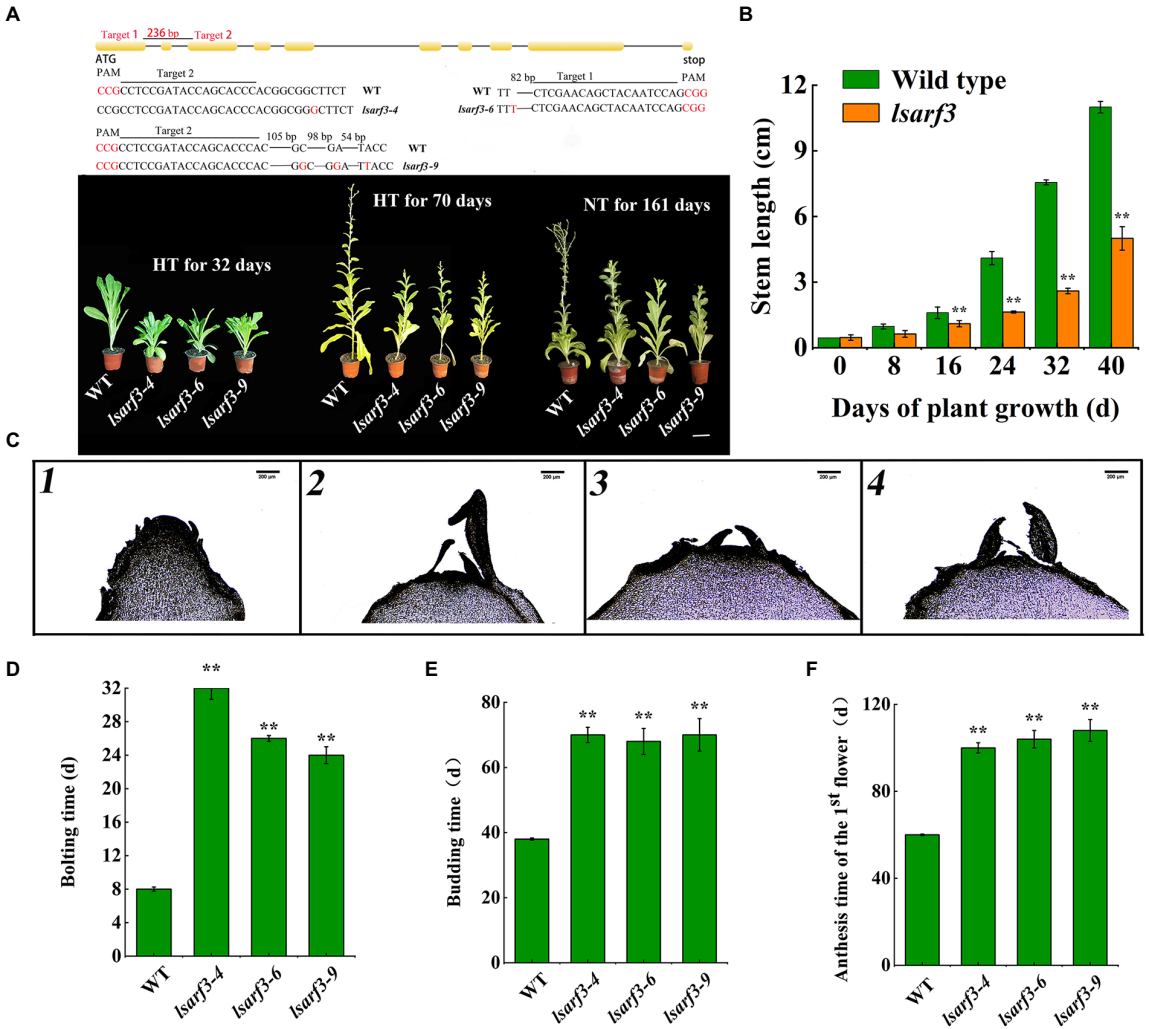
Knockout of *LsARF3* in lettuce *via* CRISPR-Cas9. **(A)** Phenotypes of WT and *lsarf3* mutant lines growing in high temperature condition for 32 and 70 days, and in normal temperature condition for 161 days, Bar = 6 cm. HT represents high temperature condition. **(B)** Stem length change of WT and *lsarf3* mutant lines. **(C)** Flower bud differentiation of stem apex in high temperature condition for 32 days. 1.WT; 2. *lsarf3-4*; *lsarf3-6*; 3. *lsarf3-9.* Bar =200 μm. **(D)** Bolting time of WT and *lsarf3* mutants. **(E)** Budding time of WT and *lsarf3* mutants. **(F)** Anthesis time of the 1st flower in WT and *lsarf3* mutants. Significant differences were tested by Student’s t-test (^**^indicates *p* < 0.01).

To further verify the *LsARF3* function, the CRISPR/Cas9 system was used to knock out *LsARF3* in lettuce. Two single-guide RNAs were designed for knockout of the *LsARF3* genes, and they were precisely targeted to the first or the second exons of *LsARF3*. Twelve T_0_ transgenic lines were identified by PCR using Cas9 gene-specific primers. Sequencing of *LsARF3* gene of T_1_ plants identified three lines of homozygous mutants, *lsarf3*-4, *lsarf3*-6, *lsarf3*-9, for phenotypic analysis. As shown in the Figure 2A, the *lsarf3-4* carries a C to G mutation at the first exon, the *lsarf3-6* harbors *a* 82-bp deletion at the first exon, and *lsarf3-9* brings insertions and deletions in the second exon. All those changes in the amino acid sequence could result in the frameshift mutations in the LsARF3 proteins. As shown in the Figure 2A, three lines of *lsarf3* mutants delayed bolting upon high temperatures. After grown in normal conditions, WT lettuce started anthesis at 161 days post germination (DPG), while lsarf3 mutants did not finished the floral transition yet (Figure 2A), suggesting that LsARF3 might play vital role in lettuce bolting and flowering. In previous studies, we have demonstrated that the GB-30 lettuce initiates stem elongation after 8-day heat treatments (Hao et al., 2018). In this study, the stem lengths of *lsarf3* mutants were significant lower than that of WT from 16 days after high-temperature treatment (Figure 2B). Microscopic observation suggested that *lsarf3* mutants entered the initial stage of flower bud differentiation after high-temperature treatment for 32 days (Figure 2C), suggesting that *lsarf3* mutants had started bolting approximately after 32-day heat treatments. Measurements of bolting time, budding time, and anthesis time of the first flower indicated that *lsarf3* mutants were about 20, 32, and 48 days later than WT plants, respectively (Figures 2D–F). At the same time, we observed the budding time of mutant plants at normal temperature, and found that it began to bud about 161 days, about 20 days later than WT (Figure 2A). Together, the results indicated that absence of functional LsARF3 postponed floral transition in the knockout lines, resulting in delayed bolting and flowering under normal or heat conditions.

### Overexpression of *LsARF3* gene accelerates bolting of lettuce

**Figure 3:**
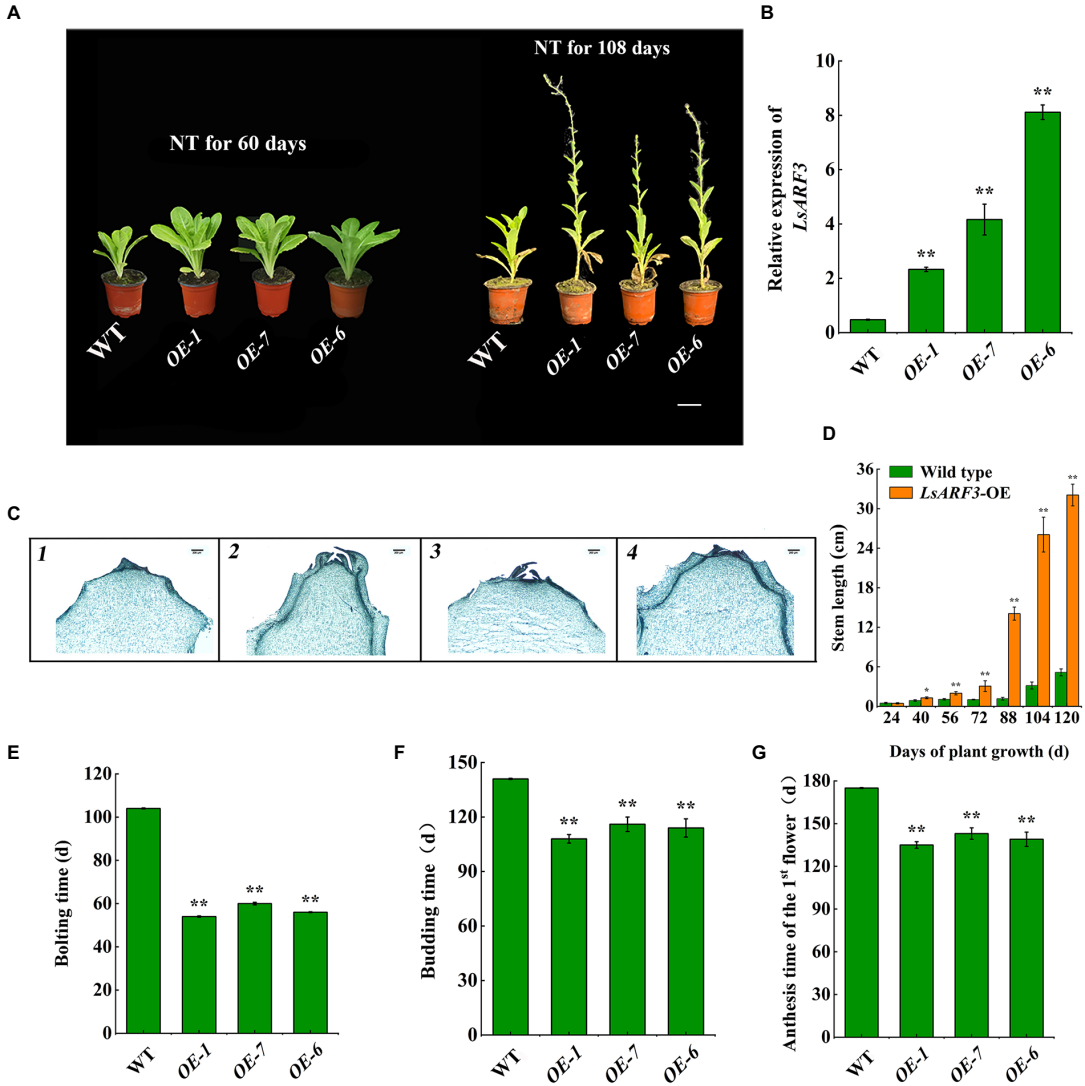
Overexpression of *LsARF3* in lettuce. **(A)** Phenotypes of WT and *LsARF3*-OE lines. Bar = 6 cm. NT represents normal temperature condition. **(B)** Expression level of *LsARF3* in WT and *LsARF3*-OE lines. **(C)** Flower bud differentiation of stem apex at 60 days during growing. 1.WT; 2. OE-1; OE-7; 3. OE-6. Bar =200 μm. **(D)** Stem length change of WT and *LsARF3*-OE lines. **(E)** Bolting time of WT and *LsARF3*-OE lines. **(F)** The budding time of WT and *LsARF3*-OE lines. **(G)** Anthesis time of the 1st flower of WT and *LsARF3*-OE lines. Significant differences were tested by Student’s *t*-test (^*^indicates *p* < 0.05 and ^**^indicates *p* < 0.01).

To further investigate that the *LsARF3* roles in lettuce bolting, we generated the *LsARF3*-overexpressing (OE) transgenic lettuce plants. The *LsARF3*-OE plants displayed accelerated bolting and flowering (Figure 3A). The three OE lines (OE-1, OE-7, OE-6) revealed that the expression levels of the *LsARF3* gene were nearly 4 times, 8 times, and 16 times higher than that of wild-type plants (WT), respectively (Figure 3B). Microscopic analysis showed that *LsARF3*-OE lines began the initiation stage of flower bud differentiation at 60 days, whereas the growth cone of WT control was prominent and wrapped in the middle of the leaf primordium that showed a semicircular shape, indicating they were still in the vegetative growth stages (Figure 3C). In addition, the stem length of *LsARF3*-OE lines was higher than that of WT with a significant or extremely significant difference from growing 40 days (Figure 3D). From phenotype and microscopic observation, it also supported the observations that the *LsARF3*-OE lines had entered the bolting stage after growing for 60 days. Similarly, the bolting time, budding time, and first flower anthesis time of *LsARF3*-OE lines were 48, 33, and 40 days earlier than that of WT, respectively (Figures 3E–G), reflecting that the *LsARF3* overexpression could accelerate bolting and flowering in lettuce.

### Auxin cannot recovery the delayed bolting phenotype of *lsarf3* mutant

**Figure 4:**
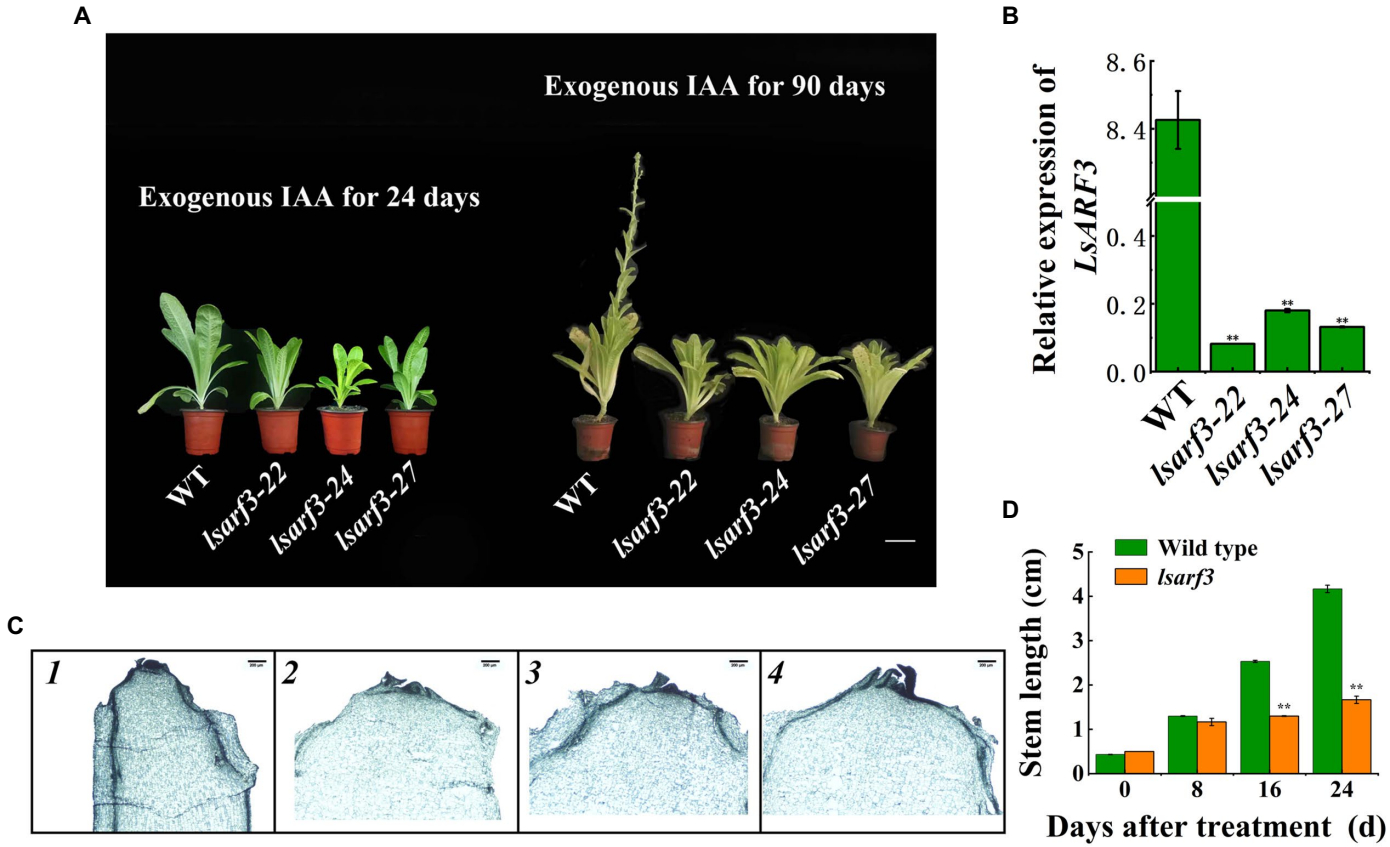
Effects of exogenous IAA on bolting and *LsARF3* expression in *lsarf3* mutants. **(A)** Plant morphological character after exogenous IAA application. **(B)** *LsARF3* gene expression after exogenous IAA application for 24 days. **(C)** Flower bud differentiation of stem apex after exogenous IAA application for 24 days. **(D)** Stem length change after exogenous IAA application. Significant differences were tested by Student’s *t*-test (^**^indicates *p* < 0.01).

Our previous studies have shown that exogenous IAA promoted bolting, and it occurred begin 8 days after exogenous application (Wang et al., 2022b). To explore whether LsARF3 was involved in auxin-induced bolting of lettuce, the exogenous IAA was applicated to WT and *lsarf3* mutants. After the application of exogenous IAA, the plants showed obvious differences (Figure 4A). It was found that the expression levels of *LsARF3* in *lsarf3* mutants was extremely significant lower than that of the WT(Figure 4B). Miscroscopy experiments demonstrated that, after exogenous IAA application for 24 days, WT plants entered the stage of flower bud differentiation, but knockout mutants were insensitive to auxin applications (Figure 4C). The stem of WT lettuce elongated rapidly after IAA application, while it was not observed in the *lsarf3* mutants. After 24 days after IAA application, the stem lengths of *lsarf3* mutants were shorter by 58.3% to that of the WT (Figure 4D). Thus, it could be concluded that WT plants started bolting on 24th Day after IAA application, but at that time, *lsarf3* mutants were still in the vegetative stage. Afterward, the stems become visible and elongated above the rosette leaves in WT plants; in contrast, the stem of *lsarf3* mutants is still embedded in the rosette leaves of transgenic lettuces. It took 90 days to entered budding stage for WT lettuces, whereas *lsarf3* lines kept at vegetative stages (Figure 4A). In other words, the defects in bolting time of *lsarf3* mutants could not fixed by exogenous IAA applications (Figures 2A,E, 4A). Together, the results suggested that exogenous auxin accelerated bolting in WT, but it cannot recover the delayed bolting phenotype of *lsarf3* mutants.

### The *LsARF3* positively regulated the expression of flowering-related genes

**Figure 5:**
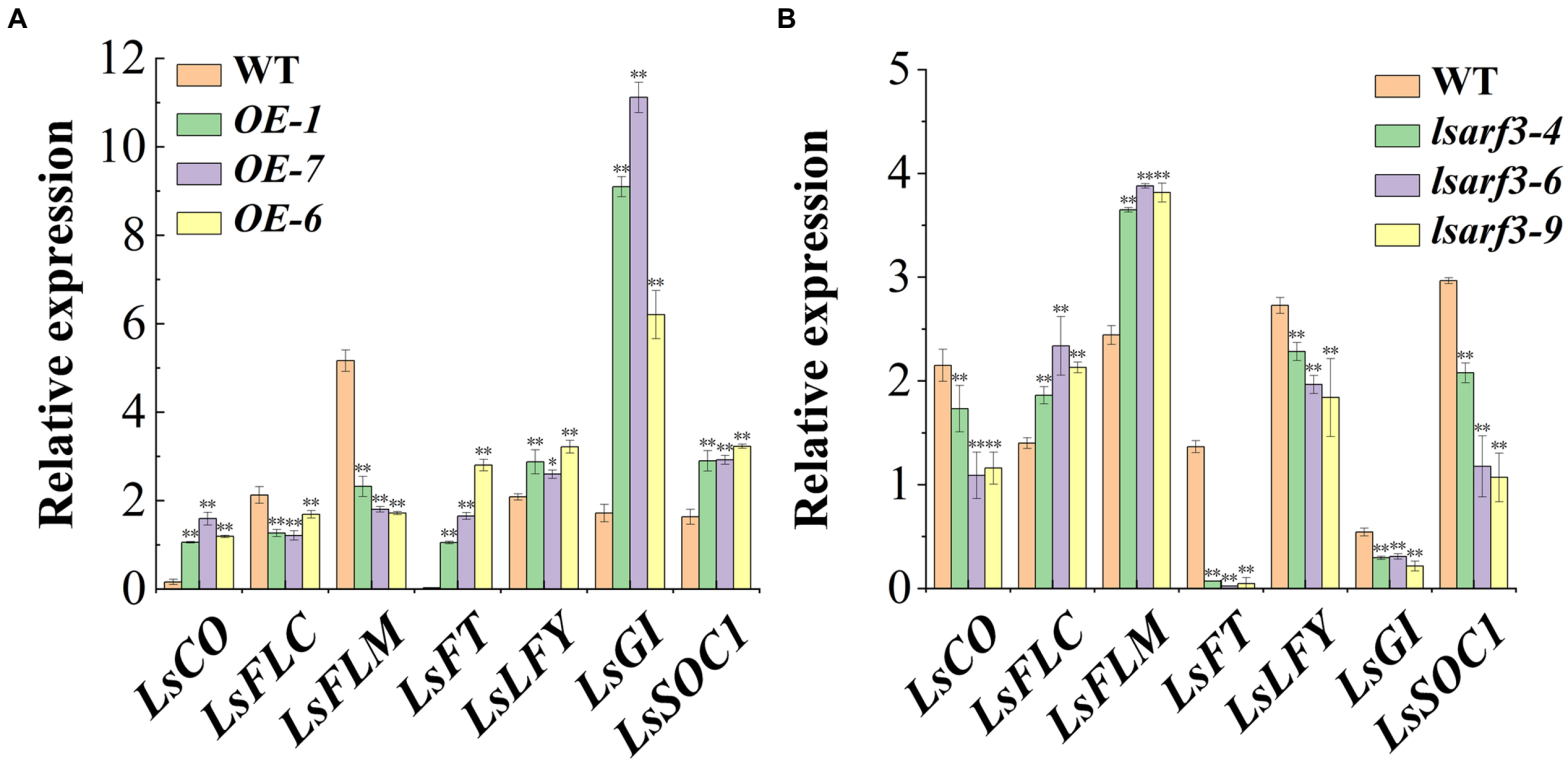
Transcript levels of flowing related genes in WT, *LsARF3*-OE and *lsarf3* mutants. **(A)** Expression levels of flowering related genes in WT and *LsARF3*-OE lines. **(B)** Expression levels of flowering related genes in the WT and *lsarf3* mutants. Significant differences were tested by Student’s *t*-test (^*^indicates *p* < 0.05 and ^**^indicates *p* < 0.01).

To explore the molecular mechanism underlying *LsARF3*-mediated bolting upon thermal treatments, the expression profiles of selected flowering-related genes in *LsARF3*-OE and mutant lines were analyzed in the shoot apex samples from three *lsarf3* mutant lines and wild type plants. The results showed that the expression levels of *LsCO*, *LsFT*, *LsLFY, LsGI*, and *LsSOC1* were significantly upregulated in these three *LsARF3*-OE lines, while their expression levels of *LsFLC* and *LsFLM* were much lower compared to that of WT lettuce plants (Figure 5A). Conversely, the expression of *LsCO*, *LsFT*, *LsLFY, LsGI*, and *LsSOC1* were attenuated in *lsarf3* mutants, and accordingly, the transcript levels of *LsFLC* and *LsFLM* were significantly increased compared to WT plants (Figure 5B). The results suggested that *LsCO*, *LsFT*, *LsLFY*, *LsGI*, and *LsSOC1* exhibited similar expression pattern in the *LsARF3*-OE and knockout lines. Therefore, it is possible that those flowering-related genes could be the potential downstream genes of *LsARF3*, thereby controlling the floral transition and the bolting of lettuce.

### *LsARF3* was bind to the putative promoter region of *LsCO*

**Figure 6:**
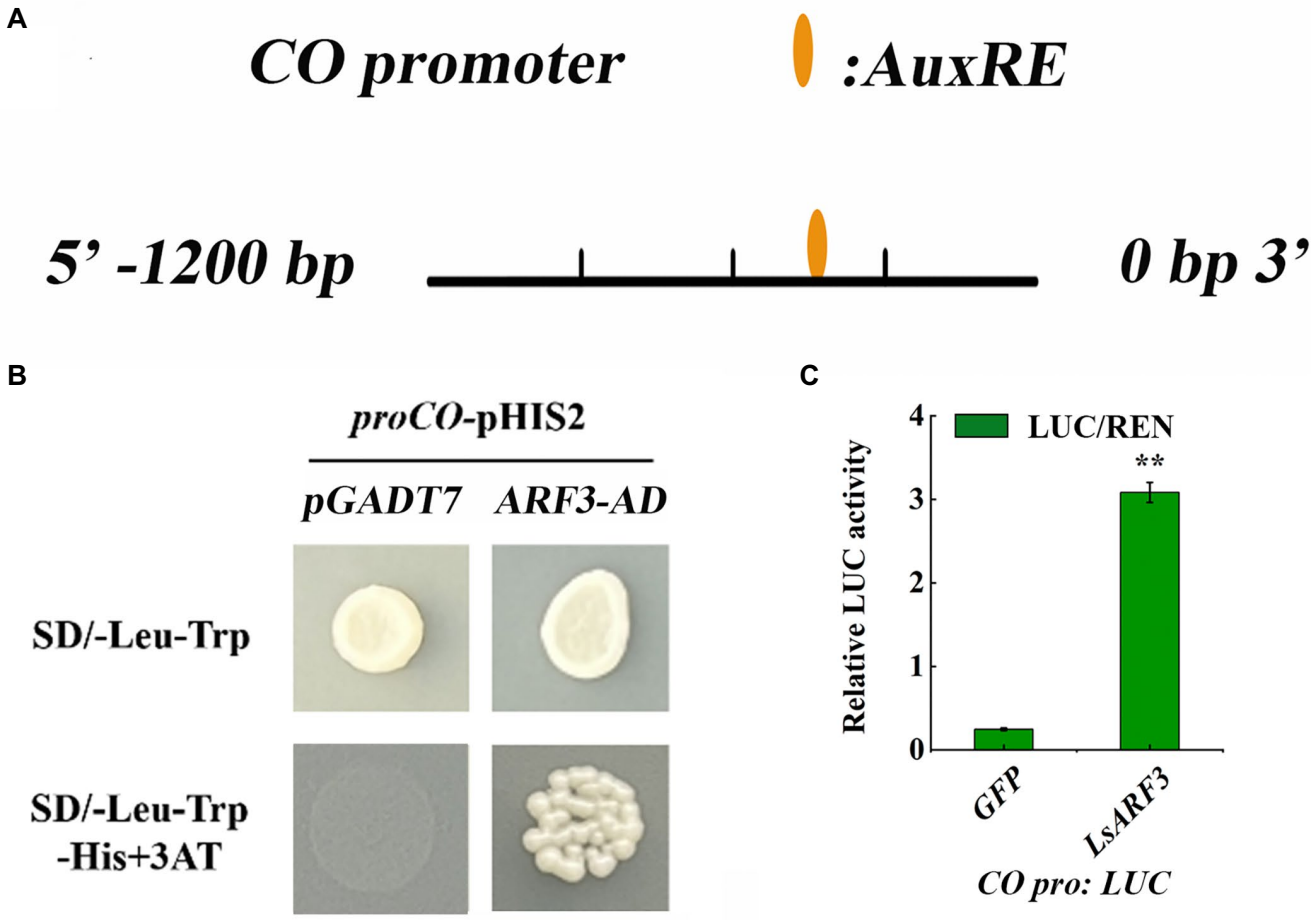
LsARF3 binds to the promoter of *LsCO* by Yeast one-Hybrid (Y1H) assay. **(A)** The AuxRE element in the promoter of *LsCO* gene. **(B)** Yeast one-hybrid (Y1H) experiment showing the binding of LsARF3 -AD to the promoter of *LsCO*. **(C)** Dual luciferase reporter assay. The GFP effector was used as a negative control, and the LUC/REN ratios of GFP were set as 1. Significant differences were tested by Student’s *t*-test (^*^indicates *p* < 0.05).

Given that *LsARF3* could co-expressed with a number of flowering-related genes such as *LsCO*, *LsFT*, and *LsGI*, we expect that the transcription of these genes might be directly regulated by LsARF3. Based on the analysis of the 2 kb promoter sequences of *LsCO*, *LsFT*, and *LsGI*, an AuxRE cis-elements in the *LsCO* promoter was identified, suggesting *LsCO* was a possible LsARF3 direct target (Figure 6A). To test the hypothesis, a yeast one-hybrid (Y1H) assay was conducted to determine whether LsARF3 can bind directly to the *LsCO* promoter. As shown in Figure 6B, the Y1H results showed that the yeast cells containing the bait vector combined with the promoter region of *LsCO* could grow on the SD/−Leu-Trp-His medium supplemented with 60 mM 3AT, when co-transformed with pGADT7-LsARF3 construct. However, the yeast cells transformed with the empty pGADT7 vector did not grow on the same selective medium. Hence, the results suggested that LsARF3 could *in vivo* bind to the promoter of *LsCO* and activate the reporter gene in yeast. This finding was further confirmed by a dual luciferase assay in tobacco leaves with *LsCO* promoter activity being monitored with a Pro*LsCO*::*LUC* reporter construct. Overexpression of LsARF3 significantly increased the activity of *LsCO* promoter (Figure 6C), further indicating that LsARF3 could promote *LsCO* expression *via* directly binding to its promoter.

## Discussion

In addition to endogenous cues, environmental variables substantially affect the floral transition among a wide range of plant species (Mcclung et al., 2016; Nagalla et al., 2021). Earlier studies have shown that high temperatures could induce premature bolting and early flowering in lettuce (Han et al., 2021). Our experiments demonstrated that exogenous applications of auxin could promote plant bolting and expressions of some *LsARFs* were associated with bolting time, implying the important contributions of auxin and ARFs to the lettuce bolting, as well as flowering. It has been reported that ARF3 is characterized by its roles in organ polarity, gynoecium patterning, self-incompatibility, and meristem activity in *Arabidopsis*. However, whether or how ARF3 mediates plant bolting remains unknown. Here, we reveal that LsARF3 controls the lettuce bolting by regulating the expression of floral transition-related genes. Our results illustrate a novel regulator model in thermally accelerated bolting and flowering pathway, which expands our understanding of how plants control developmental events in response to high temperature.

### Auxin was implicated in the heat-induced bolting in lettuce

The transition from vegetative to reproductive growth is critical to plant development because the proper timing of flowering ensures seed production required for the survival of species (Takeno, 2016). Given that auxin regulates diverse developmental processes from phototropism, root/shoot tropisms, to apical dominance in plants (Enders and Strader, 2015), it is perhaps not surprising that auxin is involved in the transition to bolting and flowering in lettuce. It was demonstrated that, under normal growth conditions, the IAA content in lettuce stems was significantly increased upon bolting, and exogenous application of IAA could promote bolting of some lettuce cultivars after 4-day treatments (Wang et al., 2022b). Recently, another study also showed that overexpression of *LsRGL1*, encoding a functional DELLA repressor, resulted in the reduction of IAA biosynthesis, while auxin feeding restored the bolting time of *LsRGL1* overexpression lines (Wang et al., 2022a). Their observations suggested that auxin was closely involved in the lettuce bolting, but whether auxin is involved in the accelerated bolting under thermal stresses remains to be examined. In our analysis, we found that IAA treatments indeed promoted the elongation of inflorescence stems under heat stresses. Accordingly, we observed that the IAA content in lettuce stems kept increasing with the progression of heat treatments (Hao et al., 2018). Therefore, our results demonstrated that auxin, at least in part, participated the thermal stress-induced bolting in lettuce, linking abiotic auxin signaling pathways with inflorescence development under abiotic stresses.

### *LsARF3* might function as transcription activators in auxin signaling cascade

ARF3 and its orthologues are found in various angiosperms, including monocots and dicots, but so far, its roles in plant development were mainly obtained from the model plant *Arabidopsis* (Hobecker et al., 2017; Brackmann et al., 2018; Zhang et al., 2018). To our knowledge, the functions of *ARF3* orthologues in monocots such as lettuce remain largely intangible. Previous extensive evidence demonstrated that ARF3/ETTIN was involved in the regulation of organ polarity determination and pattering, gynoecium morphogenesis, self-incompatibility, flower development, and other auxin-responsive behaviors in *Arabidopsis* (Nemhauser et al., 2000; Liu et al., 2014b; Zhang et al., 2018). For example, *ettin* mutants exhibited severe defects in leaf polarity specification and gynoecium patterning (Nemhauser et al., 2000). Zhang and his collogues also reported that ARF3 regulates floral meristem determinacy by directly inhibiting the expression of *ISOPENTENYLTRANSFERASE* responsible for the rate-limiting step of cytokinin biosynthesis (Zhang et al., 2018). A recent study suggested that *Medicago truncatula* plants overexpressing *MtARF3* exhibited curling leaf margins with more serrations which resemble the phenotypes of the *palm1* mutant to a large extent; further experiments confirmed that *MtARF3* restricted spatiotemporal expression of *PALMATE-LIKE PENTAFOLIATA1* (*PALM1*) through selective interaction with the promoter of *PALM1* (Peng et al., 2017). Expression of *OsARF3* construct with altered tasi-RNA targeting sites resulted in dwarf and tufty phenotypes, irregular phyllotaxis, with rolled-up or thread-like leaves in transgenic rice lines, indicating its function and regulation are conserved in higher plants (Song et al., 2012). Taken at face value, most studies supported that *ARF3s* serves as transcriptional repressors in the auxin-regulated pathways. Nevertheless, our results indicated that overexpression of *LsARF3* promoted plant growth and early bolting and flowering under normal or heat-stress conditions. Surprisingly, no developmental defects in leaf or flower organs were observed in the *LsARF3* overexpression or knockout lines, despite the slightly reduced flower fertility, which was not reported, to our knowledge, in other *ARF3* transgenic plants yet. Furthermore, the expressions of several floral genes including *LsCO*, *LsFT*, *LsSOC1*, and *LsLFY* were positively correlated with that of *LsARF3* in the overexpression or loss-of-function lines, implying their transcription could be induced by *LsARF3* during the lettuce bolting process. Thus, it is likely that *LsARF3* was able to activate the expression of those floral integrator genes at high temperatures. Importantly, it is also worth noting that LsARF3 might function as a transcriptional activator in lettuce, whereas other ARF3 homologs reported in *Arabidopsis*, legume, or rice, act as transcriptional repressors in the auxin signaling pathways. The discrepancy between *LsARF3* and its orthologues in other plant species implies that it might participate in some auxin signaling pathways in ways different from its orthologues in other plant species.

### Unique roles of LsARF3 in heat-induced bolting in lettuce

In the last decade, several genes or quantitative trait loci (QTLs) that affect the timing of floral transition have been reported to be associated with heat-induced bolting and flowering (Hartman et al., 2013; Han et al., 2021). In *Arabidopsis*, *FT*, *SOC1*, and *CO* are major floral integrators because diverse floral induction pathways are mainly converged upon these developmental regulators (Yoo et al., 2005; Valverde, 2011; Pin and Nilsson, 2012). Accordingly, their lettuce orthologues of these floral genes were functionally characterized to unveil their potential roles in floral transition, in particular under heat stresses (Chen et al., 2017; Liu et al., 2018). Overexpression of *LsFT* and *LsSOC1* can promote flowering, whereas their knockdown by RNA interference in lettuce resulted in significantly delayed bolting and insensitivity to high temperatures (Fukuda et al., 2011; Chen et al., 2017). Although lettuce COs have not been characterized, at least one CO-like gene was found to be located within flowering QTL, *qFLT5.3*, which implies its possible contributions to implicated in bolting initiation in lettuce (Seki et al., 2020). In the present study, AuxRE element was identified in the promoter region of *LsCO* gene, suggesting that CO expression might be regulated by ARF transcription factors. Indeed, *LsCO* was constitutively activated in transgenic lettuce overexpressing the *LsARF3*; by contrast, deletion of *LsARF3* dramatically attenuated the expression level in lettuce, implying that *LsCO* might be the target of LsARF3. Consequently, upregulation of *LsARF3* promoted lettuce bolting, while its knockout lines failed to accelerate bolting despite auxin feeding plus thermal treatments. Furthermore, yeast one-hybridization analysis verified that LsARF3 could physically interact with AuxRE cis-elements of the *LsCO* promoter. Therefore, our findings reinforced the idea that LsARF3 probably directly operated the transcription of *LsCO* under flowering-inducible conditions. However, the question of whether LsARF3 directly activates the expression of *LsFT* and *LsSOC1* to facilitate bolting will be examined in future experiments.

Based on our experiments, one possible scenario for interpreting lettuce early bolting upon heat stress is to postulate an auxin-*ARF3* mediated signaling pathway: thermal treatment could activate the auxin biosynthesis and/or transport in inflorescence stem, resulting in the increase of local auxin levels and subsequent *LsARF3* transcription. Subsequently, LsARF3 might directly activate the transcription of *LsCO* and indirectly induce the expression of *LsFT* and *LsSOC1* in an auxin-dependent manner, eventually leading to the accelerated bolting and flowering in lettuce. Overall, our study provides new insights into lettuce bolting in the context of ARFs signaling and heat response. However, we cannot exclude the possibility that other ARFs might be also involved in lettuce floral transition, and further experiments are still needed to investigate the functions of *ARF* genes in this important vegetable.

## Data availability statement

The original contributions presented in the study are included in the article/Supplementary material, further inquiries can be directed to the corresponding authors.

## Author contributions

JH and YH conceived and supervised the project. YL performed the experiments and analyzed the data. NL and JH wrote the manuscript. JZ, YF, ZL, ZR, NL, and CL gave advises and edited the manuscript. All authors contributed to the article and approved the submitted version.

## Funding

This work was financially supported by the National Natural Science Foundation of China (grant no. 32072560) and the Support Plan for Spark Action on Scientific and Technological Innovation of Beijing University of Agriculture (BUA-HHXD2022003).

## Conflict of interest

The authors declare that the research was conducted in the absence of any commercial or financial relationships that could be construed as a potential conflict of interest.

## Publisher’s note

All claims expressed in this article are solely those of the authors and do not necessarily represent those of their affiliated organizations, or those of the publisher, the editors and the reviewers. Any product that may be evaluated in this article, or claim that may be made by its manufacturer, is not guaranteed or endorsed by thepublisher.

## Footnotes

^1^ http://crispr.hzau.edu.cn/CRISPR2/
