# Supplementary material for: LsARF3 mediates thermally induced bolting through promoting the expression of LsCO in lettuce (Lactuca sativa L.)
Source: Front Plant Sci. 2022 Sep 8;13:958833. doi: 10.3389/fpls.2022.958833 (PMC9498183; doi:10.3389/fpls.2022.958833)
Supplement: Supplementary file 1 [file Table_1.DOCX]

Supplemental Table 1: Primer list

| Primer name | Primer sequence |
| --- | --- |
| *LsARF3*-F | atgatgtgtggtttaatcgatctgaacacc |
| *LsARF3*-R | tcacaatccttgtacacaaccatcatttgatc |
| *Ls18S*-F | GTGAGTGAAGAAGGGCAATG |
| *Ls18S*-R | CACTTTCAACCCGATTCACC |
| *LsARF3*-OE-F | CGACGGCCAGTGCCAAGCTTATGATGTGTGGTTTAATCGATCTGAACACC |
| *LsARF3*-OE-R | ATGAATTCGGATCCGGTACCTCACAATCCTTGTACACAACCATCATTTGATC |
| pRI101-F | ACTGACGTAAGGGATGACGCAC |
| pRI101-R | GCTGAACTTGTGGCCGTTTACG |
| DT1-BsF | GCTAGAGTCGAAGTAGTGATTGCTCGAACAGCTACAATCCAGCGG |
| DT1-F0 | CTCGAACAGCTACAATCCAGCGGGTTTTAGAGCTAGAAATAGC |
| DT1-R0 | GTGGGTGCTGGTATCGGAGGCGGCAATCTCTTAGTCGACTCTAC |
| DT1-BsR | CTTGCTATTTCTAGCTCTAAAACGTGGGTGCTGGTATCGGAGGCGG |
| U626-IDF | TGTCCCAGGATTAGAATGATTAGGC |
| U629-IDR | AGCCCTCTTCTTTCGATCCATCAAC |
| GFP-F | TCCAGCAGGACCATGTGAT |
| GFP-R | AACGGCCACAAGTTCAGC |
| CRISPR-DNA-F | CTCGAACAGCTACAATCCAGCGG |
| CRISPR-DNA-R | GTGGGTGCTGGTATCGGAGGCGG |
| CO-F | TGAAAACCAATTAAGCAACGAAGTAGACCA |
| CO-R | CTCGAGCCCAGGTGGTACTAC |
